# 1435. A Comparison Study of Hand-Wash Rate of Medical Personnel Before and After Automatic Hand-Wash Detector Usage in Medical Intensive Care Unit

**DOI:** 10.1093/ofid/ofad500.1272

**Published:** 2023-11-27

**Authors:** Warinthip Mahapasuthanon, Voraphoj Nilaratanakul

**Affiliations:** Vejthani Hospital, Thailand, Krung Thep, Thailand; Division of Infectious Diseases, Department of Medicine, King Chulalongkorn Memorial Hospital, Thailand, Krung Thep, Thailand

## Abstract

**Background:**

Healthcare-associated infections affect patient safety and can lead to significant complications. Pathogens can easily spread to the surrounding environment and contaminate the hands of healthcare workers (HCWs) while performing routine activities. Hand hygiene (HH) has been emphasized as an important measure to prevent the spread of infections among patients. However, compliance with HH is still considered below acceptable thresholds.

**Methods:**

This study utilized a quasi-experimental design to compare hand hygiene compliance among HCWs before and after the implementation of an electronic HH monitoring system. After system installation and test, we performed pre-intervention phase to measure baseline rates of HH episodes using the real-time location system (RTLS). HH compliance will be observed without real-time feedback and alarm.

Phase of the study

**Figure:**
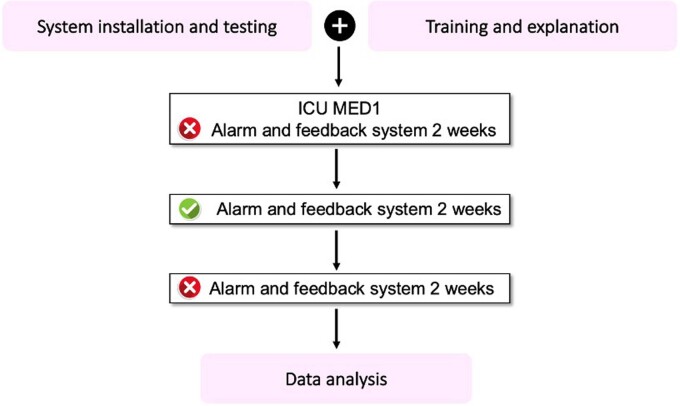

Model diagram of the medical intensive care unit (MICU) and the system

**Figure:**
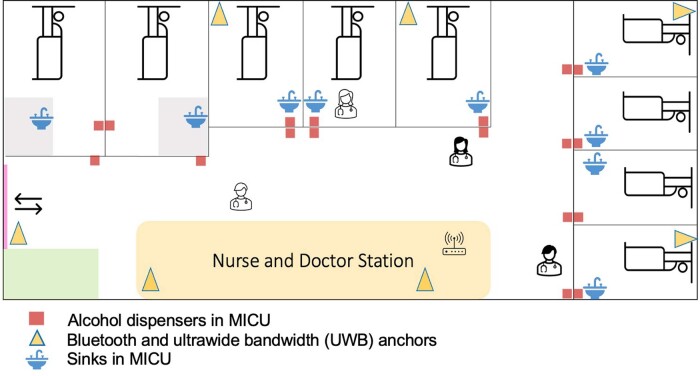

**Results:**

The sensitivity and specificity of the system that capture HH actions were 90% and 100%, respectively. For capturing HH opportunities, the system showed 100% and 90% sensitivity and specificity, respectively. During the study period, a total of 30 participants were included. All were either nurses (n=21) or nurse assistants (n=9) working in the medical ICU. 93.3% of participants were female, and almost all (96.7%) had previous experience with HH workshops. After installing the system, HH compliance was observed, and the result showed that the mean HH compliance rate before patient contact was 56.19% (95%CI, 53.27-59.99%) while the rate after patient contact was 71.06% (95%CI, 67.45-73.85%). When we compared these rates with participants’ self- estimated HH compliance, we found that system-measured HH rates were lower, with a mean difference of -16.31 (95%CI, -22.61 to -11.07, p< 0.001) and -18.94 (95%CI, -24.20 to -13.58, p< 0.001) before patient contact and after patient contact, respectively.

**Figure:**
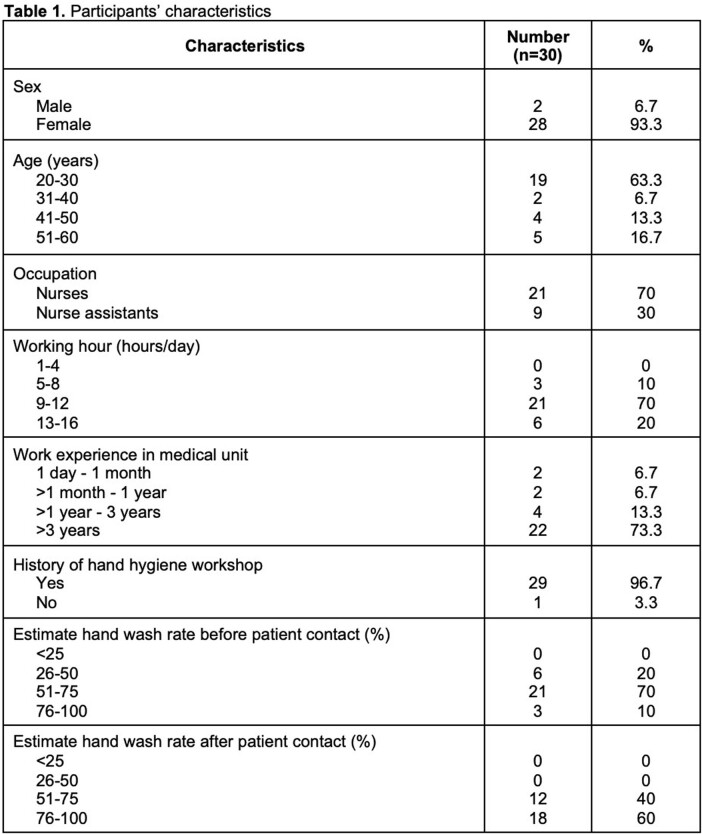

**Conclusion:**

We demonstrated a method to measure HH compliance through the implementation of an electronic HH monitoring system with real-time feedback via wireless technology. The preliminary results showed that the system based on ultra-wide bandwidth (UWB) can replace human observers. Further ongoing phases of the study will be necessary to determine whether the use of the monitoring system can effectively improve HH compliance in HCWs.

**Disclosures:**

**All Authors**: No reported disclosures

